# Identification of an intact ParaHox cluster with temporal colinearity but altered spatial colinearity in the hemichordate *Ptychodera flava*

**DOI:** 10.1186/1471-2148-13-129

**Published:** 2013-06-27

**Authors:** Tetsuro Ikuta, Yi-Chih Chen, Rossella Annunziata, Hsiu-Chi Ting, Che-huang Tung, Ryo Koyanagi, Kunifumi Tagawa, Tom Humphreys, Asao Fujiyama, Hidetoshi Saiga, Nori Satoh, Jr-Kai Yu, Maria Ina Arnone, Yi-Hsien Su

**Affiliations:** 1 Marine Genomics Unit, Okinawa Institute of Science and Technology Graduate University, Onna, Okinawa 904-0495, Japan; 2 Institute of Cellular and Organismic Biology, Academia Sinica, Nankang, Taipei 11529, Taiwan; 3 Stazione Zoologica Anton Dohrn, Napoli 80121, Italy; 4 Marine Biological Laboratory, Graduate School of Science, Hiroshima University, Hiroshima 722-0073, Japan; 5 Pacific Biomedical Research Center, University of Hawaii, Manoa, HI 96822, USA; 6 National Institute of Genetics, Yata 1111, Mishima, Shizuoka 411-8540, Japan; 7 Department of Biological Sciences, Graduate Schools of Science and Engineering, Tokyo Metropolitan University, Hachiohji, Tokyo 192-0397, Japan; 8 Institute of Biogeoscience, Japan Agency for Marine-Earth Science and Technology (JAMSTEC), 2-15 Natsushima-cho, Yokosuka 237-0061, Japan

**Keywords:** Hemichordate, *Ptychodera Flava*, ParaHox, Colinearity

## Abstract

**Background:**

ParaHox and Hox genes are thought to have evolved from a common ancestral ProtoHox cluster or from tandem duplication prior to the divergence of cnidarians and bilaterians. Similar to Hox clusters, chordate ParaHox genes including *Gsx*, *Xlox*, and *Cdx*, are clustered and their expression exhibits temporal and spatial colinearity. In non-chordate animals, however, studies on the genomic organization of ParaHox genes are limited to only a few animal taxa. Hemichordates, such as the Enteropneust acorn worms, have been used to gain insights into the origins of chordate characters. In this study, we investigated the genomic organization and expression of ParaHox genes in the indirect developing hemichordate acorn worm *Ptychodera flava*.

**Results:**

We found that *P. flava* contains an intact ParaHox cluster with a similar arrangement to that of chordates. The temporal expression order of the *P. flava* ParaHox genes is the same as that of the chordate ParaHox genes. During embryogenesis, the spatial expression pattern of *PfCdx* in the posterior endoderm represents a conserved feature similar to the expression of its orthologs in other animals. On the other hand, *PfXlox* and *PfGsx* show a novel expression pattern in the blastopore. Nevertheless, during metamorphosis, *PfXlox* and *PfCdx* are expressed in the endoderm in a spatially staggered pattern similar to the situation in chordates.

**Conclusions:**

Our study shows that *P. flava* ParaHox genes, despite forming an intact cluster, exhibit temporal colinearity but lose spatial colinearity during embryogenesis. During metamorphosis, partial spatial colinearity is retained in the transforming larva. These results strongly suggest that intact ParaHox gene clustering was retained in the deuterostome ancestor and is correlated with temporal colinearity.

## Background

The ANTP superclass homeobox genes including Hox and ParaHox encode transcription factors that play crucial roles in many aspects of development in bilaterian animals [1]. ParaHox and Hox genes are thought to have evolved from a putative common ancestral gene complex, the ProtoHox cluster, prior to the divergence of cnidarians and bilaterians [2-4]. A recent study further proposed that distinct Hox and ParaHox loci were present in the last common ancestor of all animals [5]. On the other hand, analysis on the cnidarian Hox/ParaHox genes suggested that the ParaHox cluster formed as a result of tandem duplication rather than cluster duplication [6]. One of the best-known properties of Hox genes is the spatial and temporal colinearity between their expression patterns and their positions within clusters on a chromosome [7]. Hox genes at the 3’ end are involved in patterning the anterior of the embryo, genes in the middle of the cluster pattern the central regions of the embryo, and the genes at the 5’ end pattern the posterior of the embryo in a phenomenon called spatial colinearity [8]. Temporal colinearity refers to a situation in which anterior Hox genes are expressed earlier and posterior genes are expressed later [9]. These observations have led to the hypothesis that the physical organization of Hox genes on the chromosome is important for proper morphological differentiation along the anteroposterior axis [10].

ParaHox cluster was first identified in the amphioxus *Branchiostoma floridae*, in which three member genes, *Gsx*, *Xlox*, and *Cdx*, are linked in a genomic region with *Gsx* adjacent to *Xlox* in the same orientation, followed by *Cdx* on the opposite strand [11,12]. This cluster organization is conserved in *Xenopus*, mouse, and human [12,13]. Studies on the genomic organization of ParaHox genes in non-chordate animals are limited to only a few animal taxa and the clustering seems labile. In protostomes, for example, *C. elegans* has only one *Cdx* ortholog, *pal-1*, while *Drosophila* has *Gsx* (*ind*) and *Cdx* orthologs that are not linked on the chromosome [14-16]. In the annelid *Platynereis dumerilii*, *Gsx* and *Xlox* genes are linked together, whereas *Cdx* is located away from these two genes [17]. In deuterostomes, the ParaHox genes are not linked in teleost fishes [18], the ascidian *Ciona intestinalis*[19], and the sea urchin *Strongylocentrotus purpuratus*[20], although all three genes are found. Recently, an intact ParaHox cluster was found in the starfish *Patiria miniata*[21], suggesting that ParaHox clustering was maintained in the echinoderm ancestor and the sea urchins have modified the original arrangement.

The maintenance of ParaHox clusters in chordate genomes also led to discussions about their spatial and temporal colinearity in gene expression. The expression of amphioxus ParaHox genes exhibits both temporal and spatial colinearity [11,12,22]. *AmphiCdx* transcript is the first among the three ParaHox genes to be detected by *in situ* hybridization, being expressed initially around the blastopore during mid-gastrulation and later in the larval hindgut and posterior neural tube. After gastrulation, *AmphiXlox* expression is detected in the posterior archenteron and neuroectoderm. In the larval stage, *AmphiXlox* transcripts mark the midgut-hindgut boundary. *AmphiGsx* is the last gene to be expressed, and its expression is restricted to a few cells in the anterior neural tube. Therefore, similar to the spatial colinearity of Hox genes along the anteroposterior axis, amphioxus ParaHox genes also exhibit spatial colinearity with *Gsx* expressed in the anterior neural tube, *Xlox* and *Cdx* expressed in the middle and posterior endoderm and neural tube, respectively. The temporal colinearity of amphioxus ParaHox genes is also evident, but inverted with respect to the pattern in the Hox cluster: transcript of the posterior *Cdx* is detected first and the anterior *Gsx* is expressed last [22]. Studies on the expression of ParaHox genes in other deuterostome and protostome animals including sea urchin [20], ascidian [23-26], mouse [27-29], polychaete worms [17,30,31], and gastropod [32] have also shown similar expression domains: *Gsx* genes are mostly expressed solely in the central nervous system (CNS) with a rostral anterior limit; *Xlox* genes are expressed both in the CNS and the central regions of developing guts, such as the pancreas of vertebrates; *Cdx* genes are expressed in more posterior regions of the CNS and gut. Temporal colinearity, on the other hand, is reversed in *S. purpuratus* and lost in *C. intestinalis*[33].

Hemichordates are the sister group of the echinoderms, which together are referred to as the Ambulacraria that is closely related to chordates. Hemichordates retain bilateral symmetric body plan throughout their life and share several morphological similarities with both echinoderms and chordates. Therefore, hemichordates have been served as a model to gain insights into the origins of chordate or deuterostome characters [34,35]. Hemichordates consist of two major groups, the solitary enteropneust acorn worms and the colonial pterobranchs. The phylogenetic relationships among pterobranchs and two of the enteropneust groups, direct-developing Harrimannidae and indirect-developing Ptychoderidae, have been uncertain. Fossils of both pterobranchs and enteropneusts were present in Middle Cambrian strata [36]. Phylogenetic analyses on 18S rDNA have placed pterobranchs as a sister group to the Harrimaniidae [37,38], making enteropneusts paraphyletic. A recent study that compared microRNA repertoires among several hemichordate and echinoderm species has unambiguously supported the monophyly of enteropneust worms [39]. The indirect-developing Ptychoderidae is of particular interest because their tornaria larvae share striking developmental and morphological similarities with echinoderm larvae [40-42]. *Ptychodera flava* from the Ptychoderidae has recently been shown to possess a 12-gene Hox cluster that is similar to the organization of chordate Hox clusters but with different posterior genes [43]. In this study, we investigated the genomic organization and expression of ParaHox genes in *P. flava*. We found that *P. flava* contains an intact ParaHox cluster with a similar arrangement to that of chordates. We also showed that expression of these genes exhibit temporal colinearity but lose spatial colinearity during embryogenesis. Nevertheless, partial spatial colinearity is retained in the transforming larva. These results strongly suggest that intact ParaHox gene clustering is correlated with temporal colinearity and was retained in the deuterostome ancestor.

## Results and discussion

A previous study has identified four *P. flava* ParaHox genes, which include *PfGsx*, two *Xlox* (*PfLox1* and *PfLox2*), and *PfCdx*[44]. Our PCR analysis revealed that *PfGsx*, *PfLox2*, and *PfCdx* are located on a single BAC clone, PfBS11F10. However, the presence of the *PfLox1* gene in the genome is questionable because a BLAST search of the *P. flava* genome (using shotgun sequencing; unpublished data) did not identify *PfLox1*. Moreover, several attempts to amplify *PfLox1* from genomic DNA failed, and the expression of *PfLox1* could not be detected in any developmental stages analyzed by RT-PCR and *in situ* hybridization. Furthermore, another indirect-developing hemichordate, *Balanoglossus simodensis*, contains a single *Xlox* gene (*BsXlox*) [45]. PfLox1, PfLox2, and BsXlox proteins share identical homeodomains, and the overall identity between PfLox1 and either PfLox2 or BsXlox is comparable (83% and 80%, respectively) (Additional file 1: Table S1). Thus, it is possible that, similar to other invertebrate deuterostomes [11,20,21,45], *P. flava* contains only one single *Xlox* gene (*PfLox2*). This conclusion may indicate that *PfLox1* is an ortholog gene from a related species collected in the same area as *P. flava* and that the clone was obtained from a contaminated library made from pooled animals [44]. We hence renamed *PfLox2* as *PfXlox* to represent the single *Xlox* gene in *P. flava.*

### Genomic organization of *P. flava ParaHox* genes

**Figure 1:**
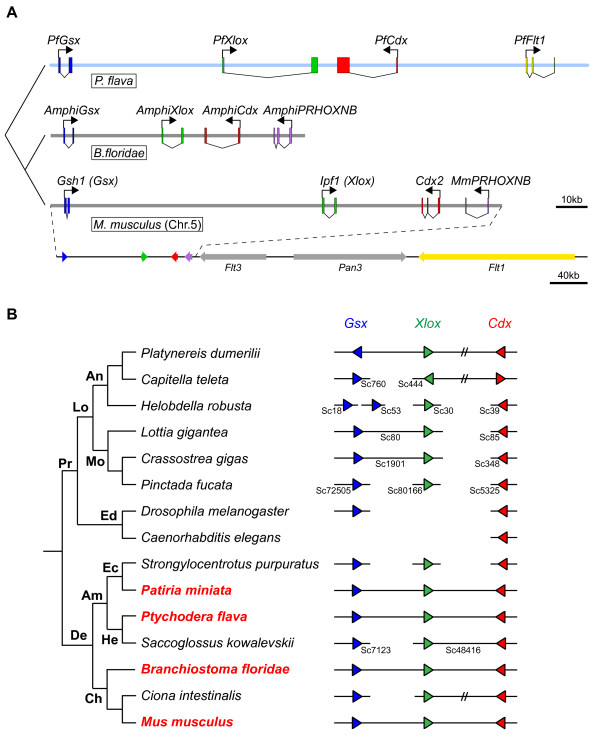
**Genomic organizations of ParaHox genes.** (**A**) Phylogenetic tree depicting genomic organization of the *P. flava* ParaHox cluster (top) compared to the clusters of amphioxus (*Branchiostoma floridae*) and mouse (*Mus musculus*) on chromosome (Chr.) 5. Blue, green, red, purple, and yellow boxes are exons of *Gsx (Gsh)*, *Xlox (Ipf1)*, *Cdx, PRHOXNB*, and *Flt1*, respectively. The light blue line indicates the fully sequenced PfBS11F10 BAC clone. Bent arrows indicate transcriptional orientations. The bottom line is a zoomed out view showing the neighboring *Flt1* gene located near the mouse ParaHox cluster. (**B**) Evolution of the ParaHox cluster in bilateria. The phylogenetic tree represents the genomic organization of ParaHox genes in several bilateria, including protostomes (Pr) and deuterostomes (De). Protostomes can be further divided into lophotrochozoa (Lo) and ecdysozoa (Ed). The following lophotrochozoa animals were included in the analysis: two polychaete species (*Platynereis dumerilii* and *Capitella teleta*) and one leech species (*Helobdella robusta*) belonging to the Phylum Annelida (An); limpet (*Lottia gigantea*) and two oyster species (*Crassostrea gigas* and *Pinctada fucata*) in the Phylum Mollusca (Mo). Two ecdysozoa species fruit fly *Drosophila melanogaster* and nematode *Caenorhabditis elegans* do not contain the full complement of ParaHox genes. In deuterostomes, Phylum Echinodermata (Ec), including sea urchin *Strongylocentrotus purpuratus* and starfish *Patiria miniata*, and Phylum Hemichordata (He), including *Ptychodera flava* and *Saccoglossus kowalevskii*, constitute Ambulacraria (Am) that is closely related to Phylum Chordata (Ch). ParaHox gene organizations of the three chordate species, amphioxus *Branchiostoma floridae*, ascidian *Ciona intestinalis*, and mouse *Mus musculus*, are presented. IDs of the scaffolds (Sc) on which ParaHox genes are located are indicated beneath the illustrated scaffolds found in the genome databases. Blue, green, and red triangles indicate the orientations of *Gsx*, *Xlox*, and *Cdx,* respectively, in the genome. Double slashes between two genes indicate that although the genes are located on the same chromosome or scaffold they are separated by intervening genes. Species names shown in red contain intact ParaHox clusters.

The sequence data of the BAC clone PfBS11F10 was assembled into a single continuous sequence of 169 kb in length (Figure 1A). *PfGsx* and *PfXlox* are organized in the same transcriptional orientation with a 47.4 kb intergenic region, while *PfCdx* is located 6.2 kb away from *PfXlox* in a head-to-head orientation. The total genomic sequence of the *P. flava* ParaHox cluster occupies a region of 106.8 kb. No other protein coding genes were identified within the cluster by routine sequence searches. This cluster organization is remarkably similar to the intact chordate ParaHox clusters found in amphioxus, *Xenopus*, mouse, and human [13]. No intact ParaHox cluster has been found outside deuterostomes. To explore the genomic organization of ParaHox genes in protostomes, we searched several recently published spiralian genomes. In the marine polychaete *Capitella teleta* genome, *Xlox* and *Cdx* are on the same scaffold with three predicted genes between them and *Gsx* is located on a different scaffold [30,46]. Two *Gsx*, one *Xlox*, and one *Cdx* genes are found in the leech *Helobdella robusta* genome, although each is located on one of four different scaffolds [46]. In the limpet *Lottia gigantea* genome, the *Gsx* and *Xlox* genes are linked together but *Cdx* is separated on a different scaffold [46]. The Pacific oyster *Crassostrea gigas* genome contains three ParaHox genes, with *Gsx* and *Xlox* located on the same scaffold and *Cdx* on another [47]. In the pearl oyster *Pinctada fucata* genome, *Gsx*, *Xlox*, and *Cdx* are located on three different scaffolds [48]. Therefore, currently there is no evidence for the existence of intact ParaHox clusters in protostome genomes (Figure 1B). Searching the draft genome of *Saccoglossus kowalevskii*, a Harrimaniidae acorn worm, revealed that while the *SkXlox* and *SkCdx* genes are next to each other in the same head-to-head orientation, *SkGsx* is on a different scaffold. It remains unknown whether the three *S. kowalevskii* ParaHox genes form a cluster in the genome. Nevertheless, the close linkage of *PfGsx*, *PfXlox*, and *PfCdx* in *P. flava* suggests that ParaHox genes were clustered in the deuterostome ancestor.

It has been suggested that the ParaHox cluster of the chordate ancestor is flanked by *CHIC* and *PRHOXNB* genes [12]. The 5’ flanking region of *PfGsx* in PfBS11F10 is too short to confirm if *CHIC* is located there in *P. flava*. Using a BLAST search against the whole genome, we identified a genomic contig containing *P. flava PRHOXNB* gene but found that this contig was not located on PfBS11F10, suggesting that the *P. flava* ParaHox cluster is not flanked by *PRHOXNB*. On the other hand, *FLT1*, the ortholog of which has been shown to be a neighbor of human and mouse ParaHox clusters [12], was found next to *PfCdx* (Figure 1A). Therefore, as in the single mammalian ParaHox cluster, genomic reorganizations have occurred around the *P. flava* ParaHox cluster but not within it.

### Temporal colinearity of *P. flava ParaHox* genes

**Figure 2:**
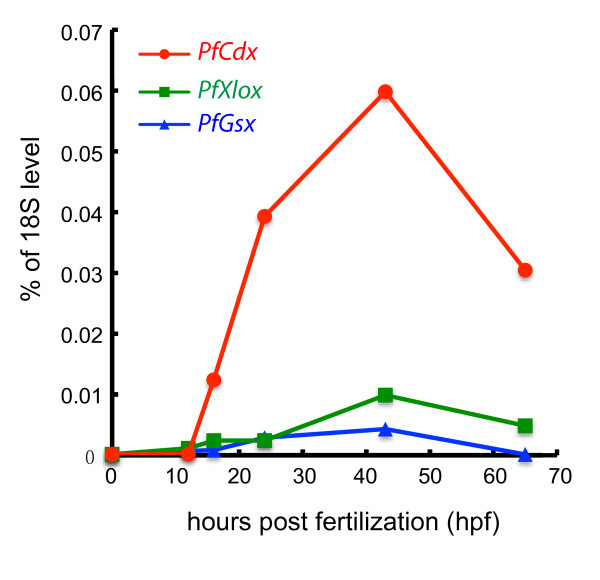
**Temporal expression profiles of ParaHox genes.** Transcript levels of ParaHox genes were measured by QPCR at different embryonic stages including unfertilized egg (0 hpf), early blastula (12 hpf), late blastula (16 hpf), early gastrula (24 hpf), late gastrula (43 hpf), and tornaria larva (65 hpf). The Y-axis is the transcript level normalized to the 18S rRNA level at the same stage.

To investigate the temporal order of *P. flava* ParaHox gene expression, we performed quantitative PCR (QPCR) at different embryonic stages (Figure 2). None of the ParaHox genes were detected in the unfertilized egg as maternal transcripts. The *PfCdx* transcript was first detected at the blastula stage (16 hours post fertilization; hpf) and peaked at the late gastrula stage (43 hpf). *PfXlox* transcript was barely detected in the blastula and early gastrula (22 hpf) stage and the expression level increased at the late gastrula stage. The *PfGsx* transcript was detected at a low level a few hours after *PfXlox* was expressed, and the transcript could not be detected at the tornaria larva stage (65 hpf). These results suggested that the first ParaHox gene to be expressed in *P. flava* is *PfCdx*, followed by *PfXlox*, and finally by *PfGsx*. As observed in amphioxus, the activation of *P. flava* ParaHox genes retains temporal colinearity.

### Spatial expression patterns of *P. flava ParaHox* genes

**Figure 3:**
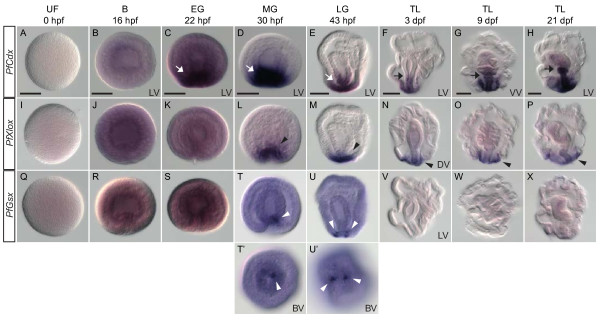
**Expression patterns of *P. flava *ParaHox genes. ***In situ* hybridization analyses of *PfCdx* (A-H), *PfXlox* (I-P), and *PfGsx* (Q-X) were performed on unfertilized egg (UF), blastula (B), early gastrula (EG), mid-gastrula (MG), late gastrula (LG), and tornaria larva (TL) at different time points (hpf, hours post fertilization; dpf, days post fertilization). White and black arrows indicate the expression of *PfCdx* and the midgut-hindgut boundary, respectively. Black and white arrowheads denote the expression of *PfXlox* and *PfGsx*, respectively. The observed views are indicated at the bottom right corner of the panels in the first row. Unless otherwise indicated, the observed views of subsequent panels are the same as the first panel in each column. DV, dorsal view; LV, lateral view; VV, ventral view. The scale bar is 50 μm. Panels **T’** and **U’** are the blastopore view (BV) of panels **T** and **U**, respectively.

**Figure 4:**
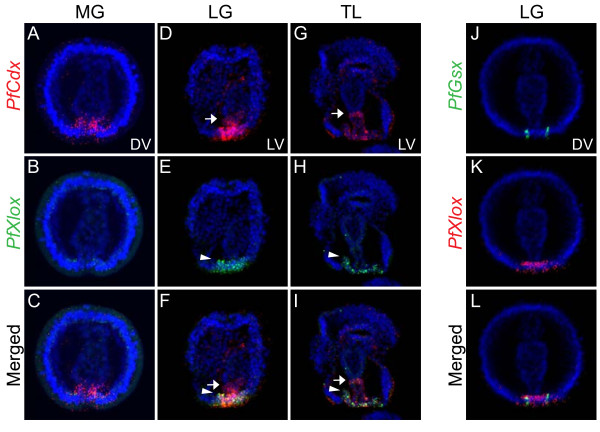
**Double fluorescent *in situ *hybridization of *PfXlox *with *PfCdx *or *PfGsx*.** DNP-labeled *PfXlox* probe and DIG-labeled *PfCdx* probe were used for *in situ* hybridization on mid-gastrula (**A**-**C**), late gastrula (**D**-**F**), and tornaria larva (**G**-**I**). The arrows and arrowheads indicate the anterior boundaries of the *PfCdx* and *PfXlox* expression domain, respectively. Double stainings of *PfGsx* and *PfXlox* transcripts were performed on the late gastrula embryos (**J**-**L**).

The spatial expression domains of the three ParaHox genes were determined by *in situ* hybridization (Figure 3). Similar to its ortholog *AmphiCdx* in amphioxus, *PfCdx* transcript was the first among the three ParaHox genes to be detected by *in situ* hybridization around the blastopore at the early gastrula stage (Figure 3C, white arrow). In the mid-gastrula stage, after the mesoderm forms at the tip of the archenteron, *PfCdx* was strongly expressed around the blastopore and the posterior archenteron (Figure 3D-E, white arrows). Later, at the tornaria larva stage, *PfCdx* expression persisted in the ectoderm around the blastopore and the hindgut until at least 21 dpf (days post fertilization) (Figure 3F-H). The anterior boundary of the *PfCdx* expression domain demarcated the midgut-hindgut boundary (Figure 3F-H, black arrows). *PfXlox* expression was detected around the blastopore at the mid- and late gastrula stage (Figure 3L-M, black arrowheads). Expression *of PfXlox* became weaker in the tornaria larva but remained in the same ectodermal domain surrounding the blastopore and the posterior endoderm (Figure 3N-P, black arrowheads). *PfGsx* expression was restricted to a few cells around the blastopore in the late gastrula stage and disappeared in the tornaria larva (Figure 3T-X, white arrowheads). To analyze spatial expression domains in detail, we performed double fluorescent *in situ* hybridization of *PfXlox* with either *PfCdx* or *PfGsx* (Figure 4). *PfCdx* transcript can be clearly observed around the blastopore at the mid-gastrula (MG) stage (Figure 4A-C). In the late gastrula embryo (LG) and tornaria larva (TL), the expression domains of *PfCdx* and *PfXlox* in the posterior ectoderm domain mostly overlapped (Figure 4D-I). *PfCdx* expression in the posterior endoderm extended to the midgut-hindgut boundary whereas *PfXlox* only expressed in the most posterior endoderm. The expression pattern of *PfCdx* in the developing gut conforms to the generally conserved feature as observed for its ortholog genes in other animals. On the other hand, *PfXlox* expression around the blastopore and the most posterior endoderm is different from the conserved expression pattern in the middle endoderm. The restricted expression of *PfGsx* also resided within the ectodermal domain around the blastopore with no detectable anterior staining (Figure 4J-L). These data indicated that expression of the *P. flava* ParaHox genes lose their spatial colinearity during the embryonic stages. The anterior boundary of the posterior *PfCdx* gene expression domain is relatively more anterior than that of the anterior *PfGsx* and middle *PfXlox* genes.

**Figure 5:**
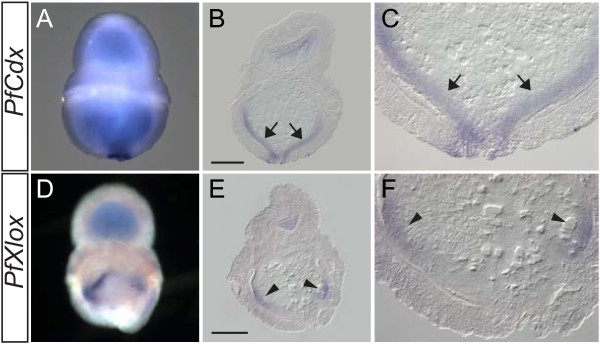
**Expression of *PfCdx *and *PfXlox *in transforming larvae.** Whole mount (**A**, **D**) and sagittal sections (**B**, **E**) of the transforming larvae hybridized with antisense probes against *PfCdx* (**A**-**C**) or *PfXlox* (**D**-**F**). Panels **C** and **F** are higher magnifications of panel **B** and **E**, respectively. Arrows and arrowheads indicate the *in situ* hybridization signals of *PfCdx* and *PfXlox*, respectively. The scale bar is 200 μm.

The adult body plan of indirect developing acorn worms, such as *P. flava,* develops from a feeding tornaria larva that undergoes metamorphosis [34]. We further investigated the expression pattern of *P. flava* ParaHox genes in the transforming larva collected from plankton tows. *PfCdx* expression was detected in the posterior endoderm (Figure 5A-C, arrows) in a pattern similar to its expression at the tornaria larva stage (Figure 3). Surprisingly, during transformation of the competent larva, *PfXlox* was expressed in the middle part of the endoderm (Figure 5D-F, arrowheads). This expression pattern is similar to that of *Xlox* genes in other animals. The *PfGsx* transcript, however, could not be detected in the transforming larva. These data indicated that the expression patterns of *PfXlox* and *PfCdx* in the endoderm of the transforming larva conform to the spatial colinearity that is conserved with other animals. Thus, we conclude that the spatial colinearity in the *P. flava* ParaHox cluster is altered during the embryonic stages, but remained in the transforming larva.

### Evolution of the *P. flava ParaHox* cluster

We have demonstrated that in the hemichordate *P. flava*, expression of the three ParaHox genes exhibits temporal colinearity during embryogenesis. However, despite having an intact ParaHox cluster, the spatially staggered expression pattern of these genes is lost in embryogenesis but partially maintained in the transforming larva. These results reinforce the idea that regulatory mechanisms controlling temporal colinearity are the major constraints that maintain an intact ParaHox cluster [7]. Spatial colinearity, on the other hand, seems to be independent of clustering. The *Cdx* expression pattern in the posterior endoderm is extremely conserved in bilateria regardless of whether *Cdx* is located within the cluster or not. Thus, the cis-regulatory module (CRM) that controls posterior endoderm expression of *Cdx* must be closely associated with *Cdx* genes during animal evolution. The embryonic expression of *PfXlox* and *PfGsx*, on the other hand, represent novel patterns. Both the conserved neuroectoderm and middle endoderm expression of *Xlox* genes are not detected in *P. flava* embryos; instead, embryonic expression of *PfXlox* is in the ectodermal cells around the blastopore. We hypothesize that this novel expression pattern is due to a new CRM generated during evolution. Compared to the genomic structures of the chordate *Xlox* genes, the relatively long intron between the two exons of the *PfXlox* gene provides the possibility for generating the novel CRM (Figure 1A). The conserved CRM controlling *PfXlox* expression in the midgut is still retained as this expression domain is maintained in the transforming larva. Further studies on the cis-regulatory analysis of *PfXlox* will be required to test this hypothesis.

A widely held view is that Hox genes are primarily used in larval cells destined to become parts of the adult body rather than in the development of larva-specific structures [33]. A similar idea, however, has not been discussed with respect to ParaHox genes. Our data show that the temporal colinearity of ParaHox genes is observed during embryogenesis whereas spatial features are more conserved in transforming larvae than during embryogenesis. These data seem to support the idea that ParaHox genes are used similarly to Hox genes in patterning anteroposterior axis for adult structures. Nevertheless, the expression of sea urchin ParaHox genes exhibits spatial colinearity in embryonic gut despite having a broken cluster [20]. Further investigation on the roles of ParaHox genes in indirect-developers may help to solve this issue.

## Conclusions

It is generally proposed that ParaHox and Hox genes have evolved from a common ancestral ProtoHox cluster. However, unlike the widespread existence of Hox clusters in the animal kingdom, intact ParaHox clusters have only been found in chordates and recently in a starfish. Here we find that the hemichordate *P. flava* contains an intact ParaHox cluster without any intervening genes and the organization of the cluster matches that of chordates and the starfish. Our finding suggests that both the ancestral Ambulacraria and ancestral deuterostome possessed intact ParaHox clusters (Figure 1B). The current status of the protostome genome assemblies could not provide definitive evidence for the presence of a scaffold containing all three ParaHox genes. Improvement of the current genome assemblies or sequencing other protostome genomes will be required to reveal whether protostomes contain intact ParaHox clusters.

The intact *P. flava* ParaHox cluster represents a special case in the discussion of temporal and spatial colinearity. The expression of these genes exhibit temporal colinearity but spatial colinearity is modified. Our data suggest that gene clustering is correlated with temporal, not spatial, colinearity. These findings may encourage more intensive studies on the regulatory mechanisms for maintaining gene clusters. These results, together with the recent finding that Hox cluster of *P. flava* has similar organization to the chordate Hox clusters [43], reinforce the idea of using *P. flava* to gain insights into the origins of deuterostome and chordate body plans.

## Methods

### Construction of BAC libraries

We constructed two sets of *Ptychodera flava* BAC libraries (PfBS and PfBH) estimated to represent the 4 (PfBS) or 5 (PfBH) × genome coverage for each library*.* BAC libraries were constructed according to the procedures as described previously [49]. Briefly, genomic DNA was prepared from sperm of a single *P. flava* individual collected at the sand bar, Kaneohe bay, Oahu, Hawaii. Genomic DNA was partially digested with *Sac* I (for PfBS) or *Hind* III (for PfBH) and subjected to pulsed-field gel electrophoresis. The DNA fragments corresponding to 110 (for PfBS) or 135 (for PfBH) kb were isolated and ligated into the pKS145 (for PfBS) or pKS200 (for PfBH) vector. Transformation was carried out by electroporation using *E. coli* DH10B as a host strain. Ampicillin-resistant transformants were collected and then stored in 384-format plates at −80°C.

### Screening of BAC libraries

To facilitate screening by PCR, BAC clones were organized in three-dimensional pools using Biomek FX. DNA was prepared from each pool using Kurabo PI-200 and PI-1100. Specific primers for each ParaHox gene were designed based on the homeobox sequences. Initially, the pools of the PfBS library were screened by PCR using *PfCdx*-specific primers and two positive clones were identified. Subsequently, DNA prepared from the isolated BAC clones was used as a template, and PCR was performed to determine the presence of *P. flava* ParaHox genes in the clones.

### BAC DNA sequencing and assembly

One *P. flava* BAC clone, PfBS11F10, containing three ParaHox genes, *PfGsx*, *PfXlox*, and *PfCdx*, was sequenced using standard shotgun procedures at the National Institute of Genetics, Japan. Individual BAC DNA was isolated using Kurabo PI-200. End sequences of the shotgun library were analyzed in an Applied Biosystems 3730xl capillary sequencer, and reads were assembled using the Phrap–Consed suite programs.

### Identification of ParaHox genes in genome databases

ParaHox gene searches were conducted using available genome databases. We used *P. flava ParaHox* sequences as queries to search the genomes of *Capitella teleta* (JGI v1.0), *Helobdella robusta* (JGI v1.0), *Lottia gigantea* (JGI v1.0), *Crassostrea gigas* (GigaDB), *Pinctada fucata* (Genome Ver 1.00), and *Saccoglossus kowalevskii* (NCBI Skow1.1).

### Animals, embryos, and larval collection

Adult acorn worms (*Ptychodera flava*) were collected from the Penghu Islands, an archipelago off the western coast of Taiwan. Animals were induced to spawn using the temperature shift method as described [50]. Eggs were washed extensively with filtered seawater before fertilization [51]. Embryos were cultured at 23°C and fed with *Rhodomonas lens* after they hatched from the fertilization envelope. Transforming larvae were collected by plankton tows at Sand Island offshore, Honolulu, Hawaii. Our experimental research was approved by Academia Sinica Biosafety Review & Biomaterials and Lab Biosafety Information System (certificate number BSF0410-00002036).

### Quantitative PCR (QPCR)

Total RNA was extracted from several embryonic stages using Trizol reagent (Invitrogen). Ten micrograms of Trizol-extracted RNA was further purified using the RNeasy Micro Kit (Qiagen) to remove genomic DNA. One microgram of the purified RNA was reverse transcribed using the iScript cDNA synthesis kit (Bio-Rad). The resulting cDNA was used as a template for QPCR. Levels of 18S rRNA were used to normalize samples. QPCR analysis was performed on a Roche LightCycler 480 with the LightCycler 480 SYBR Green I Master (Roche). The QPCR primers used in this study were designed based on the published sequences [44,52], and the primer sequences were as follows: PfGsx-QF: 5’- CGCTGTCAACAGTGCCTTAG -3′; PfGsx-QR: 5′- CACCTTCGCAGAGAGAGGAA -3′; PfXlox-QF: 5′-GGCGGACAACAAGAACACTT-3′; PfXlox-QR: 5′-CCCGCAGAAAGACTGACTTC-3′; PfCdx-QF: 5′-ACTTTCGTCACGGCAGACTT-3′; PfCdx-QR: 5′-GTCCGAATGGTGAGCTTGTT-3′; Pf18S-QF: 5′-CCTGCGGCTTAATTTGACTC-3′; Pf18S-QR: 5′-AACTAAGAACGGCCATGCAC-3′.

### *In situ* hybridization

cDNA clones of *PfGsx* and *PfCdx* were obtained from library screening [44]. PCR cloning was used to construct a *PfXlox* cDNA clone from embryonic cDNA that was amplified with PfXlox-F (5′-CCAACATGGAGAGTTCTAATCC-3′) and PfXlox-R (5′-GCGGTCTGTCTTTGTCAGAT-3′) primers. Antisense riboprobes were synthesized from these cDNA clones using digoxigenin (DIG) RNA Labeling Mix (Roche) with T7 or SP6 RNA polymerases (Promega). For double fluorescent *in situ* hybridization, dinitrophenol (DNP) labeled probes were made using the LabelIT DNP Labeling Kit (Mirus). Embryos were fixed and dehydrated as described [42]. After embryos were rehydrated in phosphate buffer saline containing 0.1% Tween-20 (PBST), they were digested with 10 μg/ml Proteinase K for 5 min, washed twice with 0.2% glycine, and fixed with 4% paraformaldehyde in PBST for 1 hr. Embryos were then washed sequentially with 0.1 M triethanolamine, 0.25% acetic anhydride, and 0.5% acetic anhydride. After washing extensively with PBST, hybridization was performed and the embryos were imaged as previously described [53]. For *in situ* hybridization of transforming larvae, similar procedures were applied except that the larvae were digested with 10 μg/ml Proteinase K for 20 to 30 min and the antibody was preabsorbed with adult powder [51]. The stained competent larvae were embedded in Tissue-Tek Optimal Cutting Temperature (O.C.T.) compound (Sakura, Japan) and cryosectioned at 10 μm.

## Abbreviations

ANTP: Antennapedia; BAC: Bacterial artificial chromosome; BLAST: Basic local alignment search tool; CNS: Central nervous system; CRM: Cis-regulatory module; DIG: Digoxigenin; DNP: Dinitrophenol; dpf: Days post fertilization; hpf: Hours post fertilization; PBST: Phosphate buffer saline with Tween-20; PCR: Polymerase chain reaction; QPCR: Quantitative polymerase chain reaction; RT-PCR: Reverse transcription-polymerase chain reaction.

## Competing interest

The authors declare that they have no competing interests.

## Authors’ contributions

YHS, MIA, JKY, HS, and NS conceived and supervised the project. TI carried out sequence analysis. YCC performed gene expression analyses. RA performed double florescent *in situ* hybridizations on the Hawaiian acorn worm embryos. HCT and CHT participated in molecular cloning and larval culture, respectively. KT and TH collected fecund animals and transforming larvae from Hawaii. RK assembled the *P. flava* draft genome. AF established the *P. flava* BAC libraries. TI and YHS wrote the manuscript. All authors read and approved the final manuscript.

## Supplementary Material

Additional file 1: Table S1 Comparison of PfLox1 to other ambulacraria Xlox proteins. Accession number: *Ptychodera flava* Lox1 (PfLox1), AY436762. *Ptychodera flava* Lox2 (PfLox2), AY436763. *Balanoglossus simodensis* Xlox (BsXlox), AB506760. *Saccoglossus kowalevskii* Xlox (SkXlox), XM_002741106. *Strongylocentrotus purpuratus* Lox (SpLox), NM_214650. Click here for file
