# Supplementary material for: Identification of an intact ParaHox cluster with temporal colinearity but altered spatial colinearity in the hemichordate Ptychodera flava
Source: BMC Evol Biol. 2013 Jun 27;13:129. doi: 10.1186/1471-2148-13-129 (PMC3698058; doi:10.1186/1471-2148-13-129)
Supplement: Additional file 1: Table S1 — Comparison of PfLox1 to other ambulacraria Xlox proteins. Accession number: Ptychodera flava Lox1 (PfLox1), AY436762. Ptychodera flava Lox2 (PfLox2), AY436763. Balanoglossus simodensis Xlox (BsXlox), AB506760. Saccoglossus kowalevskii Xlox (SkXlox), XM_002741106. Strongylocentrotus purpuratus Lox (SpLox), NM_214650. [file 1471-2148-13-129-S1.doc]

Additional file 1

**Table S1 Comparison of PfLox1 to other ambulacraria Xlox proteins.**

|  | **PfLox2** | **BsXlox** | **SkLox** | **SpLox** |
| --- | --- | --- | --- | --- |
| Homeodomain identity (%) | 100 | 100 | 99 | 97 |
| Overall identity (%) | 83 | 80 | 58 | 32 |
| Overall identity + similarity (%) | 92 | 89 | 73 | 43 |
| Indel | 3 | 6 | 12 | 15 |
